# Draft Genome Sequences of *Bacillus subtilis* Strain DKU_NT_01 Isolated from Traditional Korean Food Containing Soybean (Chung-gook-jang)

**DOI:** 10.1128/genomeA.00769-17

**Published:** 2017-08-03

**Authors:** Man-Seok Bang, Hee-Won Jeong, Yea-Jin Lee, Ha-Yeong Oh, Su Ji Lee, Moon-soo Shim, Jang-in Shin, Chung-Hun Oh

**Affiliations:** a Department of Medical Laser, Graduate School, Dankook University, Cheonan, Choongnam, Republic of Korea; b Korea Ferment, Inc., Cheonan, Choongnam, Republic of Korea; c Department of Oral Physiology, College of Dentistry, Dankook University, Cheonan, Choongnam, Republic of Korea

## Abstract

Here, we report the whole-genome sequence of *Bacillus subtilis* strain DKU_NT_01 isolated from traditional Korean food containing soybean (chung-gook-jang). The *de novo* genome of *Bacillus subtilis* strain DKU_NT_01 has one contig and G+C content of 55.4%, is 4,954,264 bp in length, and contains 5,011 coding sequences (CDSs).

## GENOME ANNOUNCEMENT

*Bacillus subtilis* is a representative Gram-positive aerobic spore-forming soil bacterium universal in the environment (1–5). This bacterium is widely used in biological experiments together with the Gram-negative bacterium *Escherichia coli*. In addition, *Bacillus subtilis* is utilized in a wide range of industrial processes as a biological control strain and main model. The beneficial effects of *Bacillus subtilis* on the balance of the intestinal microenvironment are the reason for its general use as a probiotic medication in the treatment of or prophylaxis against intestinal disorders (6–9). *Bacillus subtilis* was previously reported to be used in the manufacture of foods and has a probiotic effect (10–12). *Bacillus subtilis* strain DKU_NT_01 was isolated from traditional Korean food containing soybean (chung-gook-jang). In this study, we sequenced and assembled the genome of *Bacillus subtilis* strain DKU_NT_01 to identify genetic factors involved in high-quality production.

Total genomic DNA was extracted using the Wizard Genomic DNA purification kit (Promega, USA) according to the manufacturer’s instructions. Whole-genome sequencing of *Bacillus subtilis* strain DKU_NT_01 was performed using the Pacific Biosciences RS II sequencing platform (Pacific Biosciences, USA) at Macrogen (Seoul, Republic of Korea). The reads were assembled using the RS Hierarchical Genome Assembly Process (HGAP) protocol version 3.0, as available in subread filtering from single-molecule real-time (SMRT) Portal 2.3 (13), which generated 684,441,588 bp of data (80,967 reads; genome coverage depth, about 114). Annotation of the genomes was performed with Prokka software (14), and functional categories were predicted using RAST version 2.0 (15).

The whole-genome sequence of *Bacillus subtilis* strain DKU_NT_01 consists of a circular chromosome of 4,954,264 bp in length, with a mean G+C content of 55.4% and an *N*_50_ value of 4,954,264. The chromosome contains 5,011 coding sequences (CDSs), 88 tRNAs, and 25 rRNAs. The plasmid was not found in this strain. Genome analysis of *Bacillus subtilis* strain DKU_NT_01 revealed several gene clusters predicted to encode the biosynthesis of natural products. In particular, the genes involved in carbohydrate-related categories (including fermentation and aminosugars) were the most abundant.

Access to whole-genome sequences for these strains will enable future investigations into the possible roles that the encoded metabolites might play in high-quality production.

### Accession number(s).

The complete genome sequence of *Bacillus subtilis* strain DKU_NT_01 has been deposited in GenBank under the accession number CP021137.
